# Urban Options for Psychological Restoration: Common Strategies in Everyday Situations

**DOI:** 10.1371/journal.pone.0146213

**Published:** 2016-01-05

**Authors:** Henk Staats, Helena Jahncke, Thomas R. Herzog, Terry Hartig

**Affiliations:** 1 Department of Social and Organizational Psychology, Leiden University, Leiden, The Netherlands; 2 Department of Occupational and Public Health Sciences, University of Gävle, Gävle, Sweden; 3 Department of Psychology, Grand Valley State University, Allendale, Michigan, United States of America; 4 Institute for Housing and Urban Research, Uppsala University, Uppsala, Sweden; University of Marburg, GERMANY

## Abstract

**Objectives:**

Given the need for knowledge on the restorative potential of urban settings, we sought to estimate the effects of personal and contextual factors on preferences and restoration likelihood assessments for different urban activities-in-environments. We also sought to study the generality of these effects across different countries.

**Methods:**

We conducted a true experiment with convenience samples of university students in the Netherlands (n = 80), Sweden (n = 100), and the USA (n = 316). In each country, the experiment had a mixed design with activities-in-environments (sitting in a park, sitting in a cafe, walking in a shopping mall, walking along a busy street) manipulated within-subjects and the need for restoration (attentional fatigue, no attentional fatigue) and immediate social context (in company, alone) manipulated between-subjects. The manipulations relied on previously tested scenarios describing everyday situations that participants were instructed to remember and imagine themselves being in. For each imagined situation (activity-in-environment with antecedent fatigue condition and immediate social context), subjects provided two criterion measures: general preference and the likelihood of achieving psychological restoration.

**Results:**

The settings received different preference and restoration likelihood ratings as expected, affirming that a busy street, often used in comparisons with natural settings, is not representative of the restorative potential of urban settings. Being with a close friend and attentional fatigue both moderated ratings for specific settings. Findings of additional moderation by country of residence caution against broad generalizations regarding preferences for and the expected restorative effects of different urban settings.

**Conclusions:**

Preferences and restoration likelihood ratings for urban activity-environment combinations are subject to multiple personal and contextual determinants, including level of attentional fatigue, being alone versus in company, and broader aspects of the urban context that vary across cities and countries. Claims regarding a lack of restorative quality in urban environments are problematic.

## Introduction

Ours is an urbanizing world. As of 2010, around the globe, more people live in cities than in rural areas [1]. This trend will continue and intensify: more people will come to live in bigger cities in the coming decades. This trend inevitably has implications for activities that fulfill mundane psychological needs. Of particular interest here, activities that satisfy recurrent needs for recovery from attentional fatigue and stress increasingly will have to orient toward leisure opportunities in urban environments. Research is needed to assess the opportunities for psychological restoration that people currently find in the urban context in comparison to opportunities that people seek outside of an urban context in rural and wilder natural settings. Such research can support efforts to promote more effective restoration and so serve urban public health.

An “urban” perspective on opportunities for psychological restoration is also encouraged by recognition that the nature in focus in so much of the earlier work on restorative environments is close to an urban home or workplace and easily accessible via streets and other pathways in an urban infrastructure [2, 3, 4]. This is the case for woodlands on the urban periphery, for larger and smaller parks within cities, and for trees and other greenery planted along residential streets (for example, [5, 6, 7, 8, 9]).

In advancing an urban perspective on psychological restoration here, we extend a series of studies on how preferences for natural versus urban public environments, and judgments regarding the possibility of restoration in those environments, depend on a person’s current state of attentional fatigue, the activity the person can engage in at the time, and the immediate social context for the given activity. As in our earlier studies, we use a scenario methodology to manipulate these three factors together with environment. We thus ask our participants to remember themselves being in a situation described in a given scenario. A scenario method can only approximate the experiences that people will have when actually in the situation described, but it can nonetheless provide insights that are valuable at this early stage of research. Earlier studies in this line affirm that scenarios are a valid means of collecting evaluations of familiar situations, with results of scenario-based research subsequently affirmed by results from comparatively expensive studies of experiences in actual situations [10, 11, 12, 13]. The scenario method is thus well-suited to the goal of this study–advancing understanding of the interplay of fatigue condition, social context, and activity-in-environment within situations that are all familiar, occur frequently, and are close at hand in an urban context.

Like the earlier studies in this series, the present study uses environmental preference and restoration likelihood judgments as criterion measures, We thus use both a general indicator of appreciation and an indicator that explicitly addresses the perception of restorative potential in a given situation. Previous studies have shown that preference and restoration likelihood are strongly correlated for some categories of environments, such as small urban parks [14], but that they have distinctive patterns of environmental predictors. In other words, although people can appreciate an environment for a variety of reasons, for some kinds of environments the possibility of psychological restoration will provide an important frame of reference for preference judgments (cf. [15]).

Going beyond our earlier studies, we consider here how the influence of fatigue condition, immediate social context, and activity-in-environment on preference and restoration likelihood judgments may additionally depend on a broader societal context. In particular, we are interested in a broader context as reflected in general aspects of the urban environment, such as population density and dominant transportation mode. In the present study we address this broader societal context by looking at the interplay of attentional fatigue, social context, and activity-in-environment in three different countries. In the following sections we set out our rationale and hypotheses regarding these determinants of preference and restoration likelihood judgments.

### Activity-in-Environment

Psychological studies of urban life commonly emphasize that life in the city is more taxing than life in rural settlements [16]. Information overload [17], noise and crowding in and around the home [18], in public transportation [19], and in public places in general [20] are all more prevalent in urban than in suburban or rural environments. In a variety of ways, then, urban life imposes demands that engender a need to recover mental capabilities a person ordinarily possesses but has depleted. Here, however, we want to challenge the implicit assumption that the imposition of demands is uniform across all settings within the urban environment, and for all activities that ordinarily take place within these urban settings.

Settings and behavior are loosely related because some settings do allow great variety in behavior. But there is nonetheless a degree of correspondence: not all settings are compatible with all kinds of behavior, and some behaviors only occur in some specific settings. Relevant for our research is that some settings are meant for leisure behavior, and others are mainly meant for occupational or instrumental purposes. We think that differences found in comparisons of the restorative quality and restorative effects of urban and natural settings reflect in part on the different behavioral functions of the settings compared. Whether on site or with simulations, many studies have compared nature, and specifically settings containing a path through a natural appearing area meant for recreation, with an urban street setting clearly meant to serve transportation functions [12, 21, 22]. Both can be used for walking, but in such studies the natural setting has generally proven more restorative.

Use of an urban street setting in previous research on restorative environments has served particular purposes. A clear contrast with a natural setting has supported description of the relative restorative quality of green settings and the affective, cognitive and psychophysiological processes through which benefits of contact with nature can be realized [23]. The relatively low restorative value of urban street settings has by now been clearly demonstrated, just as concern has mounted that busy streets are being taken to represent all urban settings of relevance for restoration ([10], p.156). These facts encourage further study of the restorative potential of other urban settings.

It is certainly not evident that the strong difference found for a street versus a natural setting will be found with all other urban settings, as urban settings other than busy streets may also engender beneficial changes. In fact, different bodies of scientific literature offer many indications that particular built settings–museums [24], places of worship [25, 26], and not least homes [27, 28]–frequently function as restorative settings. Karmanov and Hamel [29] also show that a particular part of a city, in their case a recently built neighbourhood with housing along canals, can have restorative qualities comparable to a particular natural area, a nature reserve comprised of meadows, groves of trees, and farms, interspersed with creeks.

These arguments and the evidence from previous studies encourage a distinction between urban settings that are suitable for restoration and those that serve other functions. Thus, our research question is to what extent and in what circumstances specific settings within an urban environment are appreciated as settings for restoration. It seems particularly appropriate to look at settings that are familiar, close by, attractive to broad segments of the population, and frequently visited. These criteria exclude some settings; Kaplan, Bardwell and Slakter [24], for example, note that a museum may only have restorative value for experienced museum visitors, who constitute a limited segment of the population. The same holds for those on a retreat in a monastery [30].

For the present study we therefore contrast three familiar kinds of urban leisure settings–a café, a shopping mall, and a park–with a sidewalk along a busy road, previously used as the urban comparison condition in studies concerned with restorative effects of nature experiences, as mentioned above. In addition to specifying the setting, we also specify the situations under study with regard to the activity in which the person is engaged. Building on earlier work in this line [31], we thus refer to common leisure activities that our participants could normally plan to engage in while in the different environments. The activity-in-environment combinations are as follows: sitting in a café, window shopping in a mall, sitting on a bench in an urban park, and walking along a busy road. We expected differences in (a) preference expressed and (b) restoration likelihood judgments made for these activities-in-environments, with a significantly lower score for walking along a road than for the other three (Hypotheses 1a and 1b). Additional expectations regarding the relevance of activity-in-environment also take into account the social context, the person’s need for restoration (i.e., level of attentional fatigue), and interactions among the three factors across the three countries.

### Social Context

People are social creatures. The need to belong [32], to compare oneself with others [33]), and to identify with groups [34], are all commonly expressed in diverse human behaviors. It follows that transactions with physical environments reflect the social meaning that these environments can have, and that social transactions taking place in these environments may be important causes of experiences that bear on well-being, considered as satisfaction with life in general. This should also apply to environments used for restoration and the judgment of places as restorative environments, defined as settings that promote recovery from conditions such as attentional fatigue and stress [35]. Little research has been done on this topic to date, as researchers have attended primarily to the role of the physical environment in restorative processes; however, a literature on the social context of restoration is beginning to develop [11, 13, 31, 36, 37, 38]. This work acknowledges that “social context” is a broad concept, and it has narrowed the focus to distinctions between the presence versus absence of different types of persons relevant to the leisure situation, namely, family and friends that might accompany the individual versus unknown people that the individual might encounter during an outing [38]. In the research on psychological restoration, the current findings mostly pertain to effects of the company of friends, particularly in nature.

Conceivably, the presence of family or friends can serve multiple functions in the leisure situation. It may provide the opportunity for highly valued interactions [39, 40], and it may enhance interest in aspects of the setting and thus deepen the experience through common interest. Company can also enable psychological restoration, in that restoration may not proceed when the individual is alone. The most prominent enablement explanation is that company offers some assurance that the person will be safe [41]. Safety is a pervasive concern, and a lack of safety will hinder the process of restoration [42]. For example, Staats and Hartig [11] found that their study participants expressed a preference for having company while on a forest walk; however, when adjusting for experienced safety during the walk, this preference shifted and the participants preferred to walk in the forest alone. Given the many ways in which the company of family or a friend may serve psychological needs, we expect that being in the company of a family member or friend will in general increase preference for the activity-environment combinations in focus here (Hypothesis 2); however, in light of the previous research [11, 13, 31], we expect this beneficial effect of company to be more pronounced for some activities (sitting in a café, shopping in a mall; walking along a busy street) than for others (sitting on a bench in a park) (Hypothesis 3). Again, though, we anticipate that a person’s need for restoration is another factor in need of consideration, with regard both to its main effect and to its role as a moderator.

### Attentional Fatigue and the Need for Restoration

Restoration involves the recovery of psychological resources that all individuals need to meet the demands of everyday life [43, 44, 45] When a psychological resource becomes depleted, a need for restoration arises and the individual becomes motivated to enter a situation likely to bring restoration. This motivation implies a higher appreciation for opportunities for restoration.

To investigate the effect of a need for restoration on preference and restoration likelihood judgments for activities-in-environments, we build on attention restoration theory (ART) [43, 44]. A central concept in ART is the capacity to direct attention. Directed attention implies that the individual concentrates on a task, situation, or behavior and wards off other stimulation competing for attention. This enables him/her to perform the task at hand, even when that task is of itself not particularly interesting. Directed attention is a limited resource. ART assumes that the capacity to direct attention diminishes with use, because it requires effort to inhibit distractions in order to direct attention. As the capacity to direct attention diminishes, a person may commit errors on tasks, show less sensitivity to others, become irritable or impulsive, and otherwise show signs of attentional fatigue. Entering a situation that does not require directed attention permits a fatigued person to rest the inhibitory mechanism on which directed attention depends. This pause is thought to allow restoration of the capacity to direct attention, which reinstates effective functioning and enhances subjective well-being.

Attentional fatigue has been measured in several ways, including self-reports [12] and performance on tasks that require attention, including the Necker Cube Pattern Control task [46] and the Digit-Span-Backwards task [47]. Such measures can also be used to represent recovery from attentional fatigue, as with change from pretest (fatigue) to posttest (recovery) when measures are obtained before and after visits to environments thought to differ in restorative quality. Levels of attentional fatigue have also been represented as a fixed effect in experiments by comparing people who have performed activities expected to naturalistically deplete attentional capacity, such as a lengthy lecture, to people who have not performed those activities [12].

Attentional fatigue may affect preferences for the activities-in-environments we have selected to study. Based on earlier work [31], we expect that people in a state of fatigue, compared to people who are not fatigued, will express a greater preference for sitting in a park than for sitting in a cafe, shopping in a mall, and walking along a busy street (Hypothesis 4). Attentional fatigue may also affect restoration likelihood assessments for some activities-in-environments by increasing the salience of the restoration goal [12]; however, earlier work does not provide sufficient grounds for hypotheses regarding restoration likelihood assessments for the set of activities-in-environments studied here.

### Country of Residence and the Broader Societal Context

To this point our discussion has focused on mundane factors in urban life that have already received attention in efforts to understand the determination of environmental preferences and expectations regarding restoration. We do not assume however that urban life or urban areas are the same across all geo-political scales. Yet, there is little information about how patterns of restorative engagement with environments differ among people in different countries. That such differences can be found is suggested by findings of cross-national variations in environmental preferences [48, 49], assuming that preferences for some environments reflect on the possibilities they afford for restoration [15]. Several variables come to mind when considering whether people in cities within different countries may experience the same need for restoration and can resolve it in similar ways. Urban population density [16], housing style [50], tenure form (e.g., owning versus renting [51]), working habits [52], dominant transportation modes [53], popular leisure activities and opportunities to be outdoors [54], and more may differ among residents of different countries in ways that bear on the restorative potential of activities-in-environments. To explore these potential differences and get an initial impression of their importance, the current study was executed with samples from cities in three countries: the Netherlands, Sweden, and the United States. The populations of these cities ranged from ca. 50,000 to 200,000 people. The cities reflect differences among the countries in characteristics such as urban population density (high in the Netherlands, intermediate in Sweden, low in the USA) and dependence on automobile transportation for everyday travel within cities (low in the Netherlands, intermediate in Sweden, high in the USA). Such characteristics can plausibly affect preferences and restoration likelihood judgments for different activities-in-environments.

### Objectives and Specific Expectations of this Study

In sum, we argue that there are good reasons to study urban settings that can provide leisure experiences which afford restoration and which are preferred by people who are in need of restoration. We see an unmet need for studying restorative experiences in an urban context, including those available in urban nature. Based on our reasoning and review of the literature, we have formulated the following hypotheses:

- Hypotheses 1a and 1b: A walk along a busy road will be (a) preferred less and (b) seen as less likely to support restoration than sitting in an urban park, sitting in a café or window shopping in a mall.
- Hypotheses 2a and 2b: Being in the company of a relative or friend will in general increase (a) preference and (b) restoration likelihood judgments for the activity-in-environment combinations under study.
- Hypothesis 3a and 3b: The company of a friend will increase (a) preference and (b) restoration likelihood judgments for shopping in a mall, sitting in a café, and walking along a busy street relative to sitting on a bench in a park.
- Hypothesis 4: A state of attentional fatigue, versus not being fatigued, will increase preference (a) and restoration likelihood (b) more for sitting in a park, than it will for sitting in a café, walking in a shopping mall, and walking along a busy road.

It is an open question to what extent the broader societal context, here referred to in terms of country of residence, will affect preference and restoration likelihood judgments and further modify the effects of the other individual and contextual determinants. Note that country of residence should be taken as shorthand for a constellation of urban characteristics and ways of engaging with the urban environment (e.g., through transportation modes).

## Methods

### Ethics statement

#### Dutch study

The Leiden University Psychology Ethics Committee approved the Dutch portion of the study, including the study protocol and consent procedure. Participants provided written informed consent to participate, and their data were anonymized.

#### Swedish study

Participants were treated according to conventional ethical standards, and the study protocol complied with ethical regulations at the host institution. The subjects began the experiment with information about what they would be doing. They were advised that their participation was voluntary and could be discontinued at any time they wished without penalty, and that their data would be anonymized. They provided informed consent verbally; it was not recorded in any way, but it was a precondition for participation.

#### American study

The Human Research Review Committee at Grand Valley State University gave the study its formal approval. The consent of the participants was recorded using a standard informed-consent form, and their data were anonymized.

### Experimental Design

The design of focal interest was the same in each of the three experiments. We crossed antecedent fatigue condition (attentional fatigue, no attentional fatigue) with company (company, alone) and country of residence (Dutch, Swedish, American) as between-subjects factors. The different activities-in-environments (sitting in a park, sitting in a cafe, walking in a shopping mall, walking along a busy street) were treated as levels of a within-subjects factor (hereinafter referred to simply as settings).

### Participants and Procedures

All three samples consisted of university students, with females being in the majority in similar proportions (60–71%) in the three countries. In all three studies the procedure consisted primarily of having the participants complete the research instrument; however, randomization strategies, other aspects of the procedures, and some characteristics of the samples differed across studies, as will be described next.

#### Dutch sample and procedures

The Dutch sample consisted of 55 female and 25 male students at Leiden University. They ranged in age from 17 to 48 years (*M* = 19.9, *SD* = 4.87). They were recruited from a course in the first year of the four-year psychology program, and each one received course credit in compensation.

The experiment was administered via computer on an individual basis. The computer was programmed to randomly assign each participant to one of the four Fatigue by Company conditions. Each condition received 20 participants, and the proportions of men and women were approximately equal across the conditions. The computer program also generated a random order of presentation for the four settings for each participant.

Data collection was done during working hours on weekdays. Participants were seated in cubicles in a psychology laboratory. They received information about the research and provided informed consent. They provided data for an unrelated study in social psychology before turning to the computerized presentation of the research instrument for the present study. Participants could read the text, instructions, and rating scales displayed via the computer screen at their own pace. They were instructed to click on the ‘next page’ button when they had finished reading instructions or completing rating scales on a page. The next page then showed immediately. This procedure was followed throughout the experiment.

#### Swedish sample and procedures

The Swedish sample consisted of 60 female and 40 male students at Dalarna University College in Falun. They ranged in age from 19 to 54 years (*M* = 26.2, *SD* = 7.56). They were recruited through information posted in classrooms and corridors at the campuses in Falun and Borlänge. The students followed different major study lines, the most common of which were social care, business economics, teaching, and social science. None of them studied a subject particularly concerned with the environment generally or urban design and architecture more specifically. A film ticket was given as compensation.

The experiment was administered on a group basis with paper copies of the research instrument (i.e., a booklet). This had implications for randomization procedures. A random number generator was used to obtain two setting orders (order 1 –café, mall, park, street; order 2 –park, street, mall, café). Both setting orders were used in roughly equal numbers in the four versions of the booklet. Each version of the booklet represented one of the Fatigue by Company conditions. Thus, there were eight different versions of the booklet, one for each cell of a 2 (Fatigue) x 2 (Company) x 2 (Setting order) between-subjects design. Two constraints guided the distribution of booklets: each set of eight booklets should represent one complete replication of the between-subjects design, and there should be similar proportions of men and women in each cell of the design. To meet the latter constraint, separate sets of booklets were used for distribution to the men and women. With these procedures, 24 to 26 participants were assigned to each cell of the Fatigue by Company design.

The 31 data collection sessions were held in campus classrooms during normal working hours on weekdays. Each session included one to six students. They first received information about voluntary participation and the general setup of the procedure they would go through. They then provided informed consent and completed the given booklet.

#### American sample and procedures

The American sample consisted of 316 university undergraduates at Grand Valley State University, Allendale, Michigan (208 females, 81 males, and 27 who did not report their gender). The vast majority, 243 (77%), were less than 20 years old. Another 45 were in the 20–24 age range, 3 more were 25 or above, and 25 did not report their age. All were participating as a requirement for a course in introductory psychology. Each received course credit in compensation.

As in the Swedish study, the experiment was administered on a group basis. Two random setting orders were created (order 1 –café, mall, park, street; order 2 –mall, park, street, café) and used for each of the four between-subjects conditions. In each data collection session the distribution of booklets was subject to the constraint that each set of eight booklets represented one complete replication of the between-subjects design (i.e., two fatigue conditions by two company conditions by two random orders of presentation for the four settings). With these procedures, 78 to 81 participants were assigned to each cell of the Fatigue by Company design.

The 15 data collection sessions were held in the same classroom during normal working hours on weekdays. Each session included nine to 34 students. Participants first received information about voluntary participation and the general setup of the procedure they would go through. They then provided informed consent and completed the given booklet.

#### Differences among samples in participant characteristics

The three samples were similar in terms of the gender division (p > .60). After recoding the age data for the Swedish and Dutch samples to correspond to the categories used for the American samples, we affirmed the impression from the descriptive statistics that the Swedish sample was older than the other two samples (chi-square = 215.6, df = 4, p < .001). Although we cannot confirm it statistically, the Swedish sample also likely distinguished itself in terms of subject of study, in that it included students from diverse lines of study while the Dutch and American samples both consisted of introductory psychology students. Although they may have followed different lines of study, all participants shared the same occupation, and so experienced similar working demands that would contribute to restoration needs as represented in the fatigue scenarios.

### Instrument

The research instrument was developed for this set of studies, and the material was organized similarly in each one. It was first developed and used for the Dutch study, in which it was presented via computer. It was then translated into English and that version was then translated into Swedish, in both languages for presentation in booklet form. Repeated rounds of consultation among the research teams ensured that the instructions and items had the same essential content across the languages.

As already indicated, different versions of the instrument were used with each sample, whether presented via computer or in booklet form. Each version had a different order of presentation for the settings of interest. All versions had the same major components, however, and these were arranged in the same order for each sample. Those relevant here were as follows: (1) the scenario for the given fatigue condition (attentional fatigue or no attentional fatigue); (2) items about experiencing the given fatigue condition (a manipulation check); (3) the scenario for the given company condition (alone or with a friend), together with instructions about the subsequent presentation of the four settings (i.e., activities-in-environments); (4) a brief description of the first of the settings, together with reminders about the given fatigue and company scenarios; and (5) items about preference and likelihood of recovery, also with reminders about the given fatigue and company scenarios. The latter two components were then repeated for the remaining three settings. Finally, (6) the participants answered questions about the ease of following the given scenarios and other aspects of the procedure, as well questions about gender and age. In the next two subsections, we first describe the different scenarios used to represent experimental factors and then the measures used to address our research questions and checks our manipulations.

#### Antecedent condition scenarios

We first manipulated the condition antecedent to “entering” the different settings by asking the participants to follow a scenario in imagining themselves as being either attentionally fatigued or completely free from attentional fatigue. We used the same scenarios used in the study by Staats and Hartig [11]. For the attentional fatigue scenario, the text read as follows:

“The period between semesters has been very relaxing. You really had the time to recover. You feel refreshed and very energetic. You feel very much able to focus on your courses again.”

For attentional fatigue we used this text:

“This semester you have studied intensely. Now, at the end of the week of exams, you really have had it. You have difficulty concentrating and are very irritable.”

#### Company scenarios

We manipulated company by asking the participants to follow a given scenario in imagining themselves as being alone or in the company of a close friend when in the given setting. There was a long and short version of this scenario. The long version was shown once just before the presentation of the first of the four urban settings. For the in-company condition the long version read as follows:

“Now you will be given a description of four different environments where you must imagine yourself spending an hour. You are there in the company of a good friend, someone who understands you and with whom you feel at ease.”

For the being-alone condition the long version of the scenario read as follows:

“Now you will be given a description of four different environments where you must imagine yourself spending an hour. You are there alone, without anyone you know.”

In subsequent presentations of the given scenario for the remaining settings, we used a short version: “You are here in company with a good friend,” or “You are here alone.”

For each of the company scenarios, additional instructions reminded the participant to keep in mind the given antecedent condition of fatigue or no fatigue (“Try to imagine your self being in the mood described earlier”).

#### Urban setting scenarios

After the presentation of the fatigue and company scenarios, the participants were told that they would be presented with a series of four urban settings, and that they were to imagine themselves being there for one hour. Each scenario referred not only to social and physical aspects of the environment, but also to a specific activity. For the city park setting the scenario read as follows:

“You are sitting on a bench in a tree-filled park close to the center of town. There are only a few people in the area, reading or walking by. You can hear the wind stirring through the tree leaves and birds chattering. You expect to be there for an hour.”

For the café setting the scenario read as follows:

“You are sitting in a café downtown. The place has lots of people, conversing and reading, but is not packed, and there is music, not very loud. You expect to be there for an hour.”

For the shopping mall setting the scenario read as follows:

“You are walking in a shopping mall. The place has lots of people but is not packed, and there is soft background music. The mall has many different kinds of shops. You expect to be there for an hour.”

For the busy street setting the scenario read as follows:

“You are walking along an urban street which has very heavy automobile traffic. There are few people on the sidewalk. The sound from the automobile traffic is loud. You expect to be there for an hour.”

Each of these scenarios was accompanied by the given fatigue and company scenarios and was followed by a set of ratings. An example of one complete set of instructions giving fatigue condition, social context and setting is the following:

“The period between semesters has been very relaxing. You really had the time to recover. You feel refreshed and very energetic. You feel very much able to focus on your courses again.

You are sitting in a café downtown. The place has lots of people, conversing and reading, but it is not packed, and there is music, not very loud. You expect to be there for one hour.

You are there in the company of a good friend.”

### Measures

#### Checks on the fatigue scenarios

After first reading the fatigue scenario, and before reading the other scenarios, the participants were asked to describe how they would feel and behave in the antecedent condition of attentional fatigue or no attentional fatigue. They were to complete four items for affective state (feeling irritated, tired, worn out, mentally exhausted) and four items describing behaviors (would you be able to make a well-balanced decision, concentrate, foresee the implications of a complex situation, pay attention to a long lecture). This served as a check on whether the participants had understood and could follow the given scenario manipulation. The scales for the items ranged from 1 (not at all) to 7 (very much). The score used for analysis was the mean response, calculated after reversing the coding of responses to the behavioral items. The scale score could thus range from 1 (no attentional fatigue) to 7 (extreme attentional fatigue). The eight items constituted an internally consistent scale for each sample (Cronbach’s alpha = .84, .75, and .95 for the Dutch, Swedish, and American samples, respectively).

#### Ratings for the settings on preference and recovery likelihood

For each setting, preference was measured with ratings of three evaluative items, all used in previous studies in this line of research with good demonstrated reliability (see, for example [11]). The items were introduced with a statement that varied somewhat across the versions of the instrument used with the different samples. The Dutch versions of the computerized instrument introduced the items with the following statement: If I feel like this, I find being in this environment for one hour. . . (items: pleasant, annoying, attractive). With ‘like this’, the statement refers to the given fatigue condition, about which the participant had just been reminded, together with the given company scenario. In the Swedish versions of the paper booklet, the preference items were introduced with statements tailored to the given fatigue and company conditions: If I feel [EITHER … rested, energetic and capable of focusing OR … exhausted and find it hard to concentrate], I find it … (items: pleasant, annoying, attractive) … to be in the [given setting] for an hour [EITHER alone OR with a friend]. In the American versions of the booklet, the preference items were also introduced with statements tailored to the given fatigue and company conditions: If I feel [EITHER … refreshed, energetic, and able to focus OR … exhausted and have difficulty concentrating], I find being in this [given setting] [EITHER alone OR with a friend] … (items: pleasant, annoying, attractive). The order of the preference items was the same across the different settings and samples. With all versions, responses were given on a scale that ranged from 1 (not at all) to 7 (to a very high degree). The score for the scale was the mean of the three items, after reversal of the coding for the item ‘annoying.’ Scores could thus range from 1 (not at all positive) to 7 (very positive). The reliability coefficients (Cronbach’s alpha) for the measure were all above .70 across the settings and studies (see Table 1), meeting the conventional standard for internal consistency.

**Table 1:** Internal consistency coefficients (Cronbach’s alphas) for the measures of preference for the different urban settings and judged likelihood of restoration in each setting, broken out by the country of the sample.

		Setting			
Measure	Sample	Cafe	Mall	Park	Street
Preference	Dutch	.93	.92	.87	.91
	Swedish	.84	.88	.71	.72
	American	.73	.78	.69	.77
Restoration likelihood	Dutch	.75	.70	.71	.82
	Swedish	.82	.72	.77	.81
	American	.60	.61	.66	.56

Participants also rated the likelihood of recovery outcomes that a person might realize by spending time in the given setting. These items—coming to rest, renew energy—are not specific to attention recovery, but cover a more general sense of recovery that could be relevant given different antecedent condition scenarios. Like the preference items, the recovery likelihood items had been used previously in this line of research [11].

Participants were asked to rate how likely each of the outcomes would be when they imagined themselves in the setting under the given fatigue and company scenarios (1 = not likely at all; 7 = extremely likely). The score used for analysis was the mean of the responses to the two items. The reliability coefficients for the measure were above .70 looking across the settings for the Swedish and Dutch samples but not for the American samples, which showed lower internal consistency (see Table 1).

#### Checks on the measures and instructions

All versions of the instrument (computerized or booklet) concluded with questions that checked on aspects of the methods used, including the ease of imagining oneself in the condition described in the given fatigue scenario (1 = not at all easy; 7 = very easy); familiarity with the given fatigue condition (1 = not at all familiar; 7 = very familiar); ease of imagining oneself in each of the four settings (1 = not at all easy; 7 = very easy), and the clarity of the questions and instructions (1 = not at all easy; 7 = very easy). The respective scenarios were provided together with these items to facilitate the ratings.

### Statistical Analysis

#### Treatment of missing data

With the computerized administration of the experiment, the Dutch participants were required to complete all items in a given component of the instrument before they could proceed to the next one. Thus, no data were missing.

Three of the Swedish participants did not complete any of the items for the checks on the manipulation of the antecedent condition and the likelihood of recovery outcomes in the café and the park, perhaps because those items were presented on separate sheets in the given booklet and the participants in question missed the given sheets. In any case, no imputations were made and these participants were excluded from analyses involving those scales.

Data from the American sample were also complete for most of the measured variables. Ratings of the café and street on pleasantness were not provided by 11 (3.5%) and 10 (3.2%), respectively, of the American participants. Twenty-one participants did not complete either of the two items about the clarity of instructions and questions (6.6% of responses for each variable). Data were missing for other variables, but only for one to three cases (0.3–0.9%). Missing data for items in scales was replaced by the mean within each Fatigue by Company condition.

#### Calculation of internal consistency coefficients

To avoid distortions in coefficients due to manipulations of the independent variables, item scores were converted to standard scores before assessing reliability for the given scale (internal consistency indexed with Cronbach’s alpha). For the eight items in the check on the fatigue manipulation, this was done separately within each Fatigue condition. For the items in the preference and recovery likelihood scales used to rate each of the different settings, standard scores were generated separately within each Fatigue by Company condition. Alpha was then calculated for these standard-score scales using the data for all participants in the respective sample.

#### Tests of the effects of the experimental manipulations

The experimental design included setting as a within-subjects factor with four levels, fatigue as a between-subjects factor with two levels, and company as a between-subjects factor with two levels. Where appropriate, we report Greenhouse-Geisser corrected degrees of freedom and related p-values for within-subjects effects. We use the repeated contrasts procedure in SPSS 20.0 to assess differences between successive pairs of settings. The distributions of the preference and restoration likelihood ratings were positively or negatively skewed for the different settings. We therefore checked our conclusions using non-parametric procedures.

We considered effects of two other between-subjects factors–gender and the order in which the four settings were presented. We evaluated order effects only with the Swedish and American subjects, as the procedure was administered with individual random orders to the Dutch participants. Different sets of random presentation orders were drawn for the American and Swedish samples, so we evaluated them separately. Presentation order did not have a main effect on ratings of preference in the American sample (*p* > .98, η^2^_p_ < .001), nor did it interact with setting (*p* > .32, η^2^_p_ = .004). Presentation order did have a main effect on ratings of restoration likelihood in the American sample (*p* = .03, η^2^_p_ < .014), such that scores generally were slightly higher for that setting order in which the the park rather than the café was evaluated first; however, presentation order again did not interact with setting (*p* > .12, η^2^_p_ = .006). The results looked somewhat different for the Swedish sample. Presentation order did not have a main effect on ratings of preference (*p* > .85, η^2^_p_ < .001), but it did interact with setting (*p* = .01, η^2^_p_ = .042). Similarly, presentation order did not have a main effect on ratings of restoration likelihood (*p* > .74, η^2^_p_ = .001), but it did interact with setting (*p* = .02, η^2^_p_ = .035). For both variables, the interaction involved the evaluation of the park being less negative if it followed on evaluation of the café and mall. Note that while there is evidence of order effects, these effects were small. Given this fact, and the fact that the setting orders were balanced across conditions, we did not include them in the hypothesis tests.

Gender proved more influential than presentation order. The women in our sample generally rated the settings somewhat higher on preference (*p* = .006, η^2^_p_ = .016) and restoration likelihood (*p* < .02, η^2^_p_ = .011). The main effects were however conditioned by setting for both preference and restoration likelihood. For preference, the women rated the shopping mall more positively and the street setting more negatively than did the men (*p* < .001, η^2^_p_ = .019). For restoration likelihood, the women rated only the street more negatively than did the men (*p* < .001, η^2^_p_ = .014). Given that women outnumbered men substantially in each sample, we included gender in initial hypotheses tests to check whether it further moderated the effects of the focal independent variables. The only effects involving gender that were statistically significant were those we have just accounted for; gender did not moderate the effects of fatigue, company and/or country of residence. To simplify the presentation here, we do not report any additional results for gender.

### Checks on Manipulations and Instructions

We performed a series of checks on the experimental manipulations and the clarity of the instructions and materials. The first set of checks focused on whether the participants could follow the scenario for the fatigue condition to which they had been assigned. As shown in Table 2, the participants in each sample clearly distinguished between the low and high fatigue conditions in terms of how they anticipated feeling and behaving under the given scenario. Those in the high fatigue condition reported that under such a scenario they would in general experience negative affective states and have little ability to perform behaviors that required directed attention (see the description of the measure in section 2.3.2). The means for those in the low fatigue condition were toward the opposite end of the scale. For each sample the difference between the fatigued and fresh conditions was statistically significant (*p* < .001). Looking across the three samples, the high fatigue subjects in the Swedish sample had somewhat lower scores than the high fatigue subjects in the Dutch and American samples, though still well-above the mid-point of the scale (*p* < .001). In contrast, the subjects in the low fatigue condition in all three samples reported similarly low levels of assumed negative affect and performance incapability (*p* > .30). The company manipulation did not affect the reports regarding feelings and behavior associated with the given fatigue scenario (*p* > .30), nor did it interact with fatigue and/or country of residence (*p* > .40).

**Table 2:** Descriptive statistics (means and standard deviations) for the checks on the fatigue manipulation, broken out by sample country.

		Experience when in fatigue condition		Familiarity with fatigue condition			Ease of imagining the fatigue condition	
Sample		Low fatigue	High fatigue	Low fatigue	High fatigue	Low fatigue		High Fatigue
Dutch	*M*	1.91	5.46	4.90	5.82	5.82		6.03
	*SD*	0.68	0.73	1.53	0.81	0.81		0.80
Swedish	*M*	1.89	4.72	4.45	5.63	5.63		5.80
	*SD*	0.57	0.69	1.66	1.41	1.41		1.20
American	*M*	2.07	5.20	5.16	5.79	5.79		5.93
	*SD*	0.96	0.79	1.60	1.43	1.43		1.06

*Note*: The respective scales are as follows: 1 = no attentional fatigue, 7 = extreme attentional fatigue; 1 = not at all familiar, 7 = very familiar with the condition; 1 = not at all easy, 7 = very easy to imagine being in the condition.

The second set of checks assessed familiarity with the fatigue condition described in the given scenario. As shown in Table 2, in all three samples, the scores were on average well-above the mid-point of the scale, indicating substantial familiarity with the given fatigue condition. In the Dutch and Swedish samples, the participants in the high fatigue condition reported being more familiar with that condition than did the participants in the low fatigue condition (*p* ≤ .01). Members of the American sample reported similar levels of familiarity with their respective fatigue scenarios (*p* > .96). The national samples differed in the degree of the familiarity with the low fatigue condition, with the Swedish subjects reporting lower levels of familiarity (*p* < .02). The members of the different samples in the high fatigue condition reported comparable levels of familiarity with that condition (*p* > .12).

The third set of checks focused on whether the participants could easily imagine themselves in the condition described in the given fatigue scenario. The generally high means reported in Table 2 indicate that, in each sample, the participants in both fatigue conditions found it quite easy to imagine themselves in the given condition. The levels are comparable for both fatigue conditions in each sample (all three *p*s > .19), and there are no significant differences across the samples either among the low fatigue or the high fatigue subjects (both *p*s > .45).

The fourth set of checks addressed the familiarity with the activity-environment settings and the ease of imagining being in those settings (see Table 3). Participants reported being familiar with each of the settings but to different degrees (*p <* .*001)*, and the differences were moderated by country of residence (*p <* .*001)*. The mean familiarity scores ranged from *M* = 4.6 (*SD* = 1.9) for the street to *M* = 6.0 (*SD* = 1.4) for the mall.

**Table 3:** Descriptive statistics (means and standard deviations) for the checks on the familiarity and ease of imagining being in each activity-in-environment (setting) broken out by sample country.

		Setting			
Measure	Sample	Café *M(SD)*	Mall *M(SD)*	Park *M(SD)*	Street *M(SD)*
Familiarity	Dutch	5.31(1.6)	5.93(1.3)	5.38(1.5)	4.08(2.0)
	Swedish	5.43(1.5)	5.75(1.4)	5.04(1.6)	5.04(1.5)
	American	5.32(1.8)	6.14(1.4)	4.91(1.8)	4.64(2.0)
Ease of imagining	Dutch	5.75(1.2)	6.01(1.1)	6.03(1.0)	5.20(1.5)
	Swedish	5.94(1.1)	5.87(1.2)	5.99(1.2)	5.28(1.6)
	American	5.86(1.4)	6.03(1.2)	5.74(1.5)	4.95(1.8)

*Note*: The respective scales are as follows: 1 = not at all familiar, 7 = very familiar with the condition; 1 = not at all easy, 7 = very easy to imagine being in the condition.

Finally, the participants were also asked to rate how capable they were of imagining themselves in each activity-in-environment (see Table 3). There was a main effect for setting, (*p* < .001). Average scores across countries ran from *M* = 5.1 (*SD* = 1.7) for the street to *M* = 6.0 (*SD* = 1.2) for the mall, and the street was rated significantly lower than any of the other settings. We considered all scores high enough to have confidence in the validity of responses to our scenarios.

## Results

### Effects of the Activities-in Environments on Preference and Restoration Likelihood Ratings

In line with natural-urban comparisons in previous research, we expected that a walk along a busy road would be preferred less than sitting in an urban park, sitting in a café, or window shopping in a mall (Hypothesis 1a). This proved to be the case. Our participants regarded the street setting negatively (*M* = 2.7, *SD =* 1.3), while the other three settings were regarded neutrally (the mall; *M* = 4.7, *SD* = 1.6) or positively (the café; *M* = 5.2, *SD* = 1.3, and park; *M* = 5.9, *SD* = 1.1)(see also Table 4). This contributed to a large effect of setting in the RM-ANOVA, *F* (2.85, 1377.03) = 450.02, *p* < .001, η^2^_p_ = .482. Subsequent tests indicated that sitting in the park was preferred over sitting in the café, *t*(495) = 11.42, *p* < .001; that sitting in the café was preferred over shopping at the mall, *t*(495) = 6.26, *p* < .001; and that shopping at the mall was preferred over walking along the street, *t*(495) = 22.73, *p* < .001. Thus, while the participants did distinguish among the park, café and mall settings, the setting that deviates the most from the others is the street, while the park, café, and mall are relatively close in preference.

**Table 4:** Preference scores for the four settings (means and standard deviations) broken out for conditions of attentional fatigue, company and country.

			Park	Cafe	Mall	Street
Fatigue	Company	Country	*M(SD)*	*M(SD)*	*M(SD)*	*M(SD)*
High	Alone	Dutch	5.9 (1.1)	4.2 (1.6)	4.6 (1.6)	2.4 (1.5)
		Swedish	4.8 (1.5)	5.3 (1.0)	3.3 (1.5)	3.3 (1.9)
		American	6.0 (1.0)	4.5 (1.2)	4.0 (1.5)	2.4 (1.3)
	Friend	Dutch	6.3 (0.8)	5.7 (1.3)	5.1 (1.8)	2.4 (1.1)
		Swedish	6.0 (0.8)	5.3 (1.1)	4.0 (1.6)	2.7 (1.0)
		American	5.9 (1.1)	4.8 (1.3)	4.2 (1.4)	2.5 (1.3)
Low	Alone	Dutch	5.1 (1.4)	4.4 (1.6)	4.9 (1.6)	2.5 (1.3)
		Swedish	6.3 (0.7)	5.4 (1.1)	4.9 (1.4)	2.5 (1.1)
		American	6.1 (1.0)	5.4 (1.3)	4.9 (1.6)	2.8 (1.3)
	Friend	Dutch	6.1 (1.0)	6.0 (0.8)	6.1 (0.9)	2.8 (1.5)
		Swedish	6.2 (0.8)	6.0 (1.0)	4.9 (1.5)	2.8 (1.0)
		American	6.1 (1.1)	5.7 (1.0)	5.6 (1.2)	2.9 (1.3)

*Note*: Scores fall on a scale from 1 = not at all positive to 7 = very positive.

The results for restoration likelihood parallel those for preference, with a declining gradient from sitting in the park to walking along the street driving another large main effect of setting, *F* (2.94, 1420.07) = 445.21, *p* < .001, η^2^_p_ = .480. Subsequent tests indicated that sitting in the park had a higher mean restoration likelihood rating (*M* = 5.5, *SD* = 1.3) than sitting in the café (*M* = 4.3, *SD* = 1.4), *t*(494) = 13.79, *p* < .001; sitting at the café was rated higher than shopping at the mall (*M* = 3.1, *SD* = 1.5), *t*(1494) = 14.64, *p* < .001; and shopping at the mall was considered more likely to support restoration than walking along the street (*M* = 2.0, *SD* = 1.2), *t* (495) = 13.85, *p* < .001 (see also Table 5).

**Table 5:** Restoration likelihood scores (means and standard deviations) for the four settings broken out for conditions of attentional fatigue, company and country.

			Park	Cafe	Mall	Street
Fatigue	Company	Country	*M (SD)*	*M (SD)*	*M (SD)*	*M (SD)*
High	Alone	Dutch	5.5 (1.0)	3.6 (1.5)	3.8 (1.5)	2.2 (1.5)
		Swedish	4.2 (1.8)	4.6 (1.5)	1.9 (1.0)	3.1 (2.1)
		American	5.7 (1.1)	4.3 (1.6)	2.9 (1.4)	1.9 (1.2)
	Friend	Dutch	5.7 (1.3)	4.3 (1.6)	3.9 (1.8)	2.0 (0.9)
		Swedish	5.3 (1.3)	4.5 (1.5)	2.6 (1.4)	1.8 (1.0)
		American	5.4 (1.3)	4.4 (1.2)	3.3 (1.7)	1.9 (1.2)
Low	Alone	Dutch	4.9 (1.0)	4.3 (1.1)	3.8 (1.3)	2.1 (0.9)
		Swedish	5.7 (1.4)	4.2 (1.6)	2.6 (1.3)	1.9 (1.2)
		American	5.8 (1.3)	4.5 (1.4)	3.0 (1.6)	1.9 (1.1)
	Friend	Dutch	5.6 (1.4)	4.4 (1.1)	3.5 (1.0)	2.0 (1.2)
		Swedish	5.5 (1.3)	4.2 (1.7)	2.6 (1.2)	1.5 (0.5)
		American	5.4 (1.4)	4.3 (1.4)	3.4 (1.3)	2.1 (1.1)

*Note*: Scores fall on a scale from 1 = not likely at all to 7 = very likely.

### Effect of Immediate Social Context on Preference and Restoration Likelihood Ratings

The second set of hypothesis tests concerned the main effect of having the company of a friend versus being alone, collapsing across the four activities-in-environments. We expected that being in the company of a relative or friend would in general increase preference for an activity-in-environment (Hypothesis 2a). The results are in line with this hypothesis. Across the four settings, being in company was preferred (*M* = 4.8, *SD* = 0.73) over being alone (*M* = 4.5, *SD* = 0.75; *F*(1, 494) = 20.1, *p* < .01, η^2^_p_ = .039)(see also Table 4). We also expected that being in the company of a relative or friend would in general increase restoration likelihood judgments for the activity-environment combinations under study (Hypothesis 2b). This proved not be the case; the main effect of company was not significant, *F*(1, 493) = 0.532, *p* > .50 (see also Table 5).

### Immediate Social Context as a Moderator of the Effects of the Activities-in Environments on Preference and Restoration Likelihood Ratings

The third set of hypothesis tests concerned the moderating effect of having the company of a friend versus being alone on preference for the four settings (i.e., activities-in-environments). We expected that the company of a friend would increase preference more for shopping in a mall, sitting in a café, and walking along a busy street than for sitting on a bench in a park (Hypothesis 3a). The interactive effect of setting and company on preference was significant, *F*(3, 481) = 3.70, *p* < .05, η^2^_p_ = .023. Largely in line with our hypothesis, company was preferred over being alone for the café [*t* (494) = 4.20, *p* < .001] and the mall setting [*t*(494) = 3.80, *p* < .001], while for the park and the street setting preference for company was the same as for being alone (*p*s > .05; see Table 6).

**Table 6:** Preference scores (means and standard deviations) for the four settings when alone or in company.

	Alone	Company
	*M (SD)*	*M (SD)*
Park	5.9 (1.1)	6.0 (1.0)
Café	4.9 (1.4)	5.4 (1.2)
Mall	4.4 (1.6)	4.9 (1.5)
Street	2.6 (1.4)	2.7 (1.3)

*Note*: Scores fall on a scale from 1 = not at all positive to 7 = very positive.

We had the same expectations regarding the effects of company on likelihood of restoration (Hypothesis 3b) for the four settings. The interaction of setting and company was significant [*F*(3, 481) = 3.45, *p* < .05, η^2^_p_ = .007], encouraging further tests per setting. Expectations were partly met: company increased the rated likelihood of restoration for walking in a shopping mall [*t*(494) = 2.30, *p* < .05], and it did not change ratings of restoration likelihood for sitting in a park or walking along a busy street (*p* > .10). Against expectations, company did not change the likelihood of restoration for sitting in a café (p > .70; see Table 7).

**Table 7:** Restoration likelihood scores (means and standard deviations) for the four settings when alone or in company.

	Alone	Company
	*M (SD)*	*M (SD)*
Park	5.5 (1.3)	5.4 (1.3)
Café	4.3 (1.5)	4.4 (1.4)
Mall	3.0 (1.5)	3.3 (1.5)
Street	2.1 (1.3)	1.9 (1.0)

*Note*: Scores fall on a scale from 1 = not likely at all to 7 = very likely.

### Need for Restoration as a Moderator of the Effects of the Activities-in Environments on Preference and Restoration Likelihood Ratings

The fourth set of hypotheses focused on how the need for restoration might differently affect ratings of the four settings. There is a general effect on preference ratings confirming this possibility: the interaction effect of need for restoration and setting is significant [*F*(3, 482) = 9.28, *p* < .001 η^2^_p_ = .06]. More specifically we expected lower preferences for leisure activities that implied the presence of many people in an urban context, so we expected that when imagining a state of attentional fatigue, the preference for sitting in a park over sitting in a café and over walking in a shopping mall would increase compared to imagining a state of no fatigue (Hypothesis 4). Both specific interaction effects confirmed this hypothesis: fatigue increased the degree of preference for a park over a café [*F*(1,494) = 12.96, *p* < .001, η^2^_p_ = .03] and over a mall [*F*(1, 494) = 28.81, *p* < .001, η^2^_p_ = .06]. Attentional fatigue did not increase preference for sitting in the park over walking along a busy road, probably due to the fact that the street setting was considered so very unattractive in both conditions [*F*(1, 494) = 0.018, *p* > .80, η^2^_p_ = .00; see Table 8].

**Table 8:** Preference scores (means and standard deviations) for the four settings under low or high attentional fatigue.

	Low fatigue	High fatigue
	*M (SD)*	*M (SD)*
Park	6.0 (1.1)	5.8 (1.0)
Café	5.5 (1.2)	4.8 (1.3)
Mall	5.2 (1.5)	4.1 (1.5)
Street	2.8 (1.3)	2.6 (1.4)

*Note*: Scores fall on a scale from 1 = not at all positive to 7 = very positive.

### The Role of Country of Residence as a Broader Contextual Determinant

We explored whether the broader societal context, here referred to in terms of country of residence (hereinafter referred to as country), further modified the effects of the other individual and contextual determinants of preference and restoration likelihood judgments. To do so, we added country to the experimental factors–setting, company, need for restoration—in the RM-ANOVA with either preference or restoration likelihood as dependent variable.

Country appears to have had multiple consequences for the outcomes. With regard to Hypothesis 1a, country moderated the preferences for some of the settings [*F*(6, 966) = 6.84, *p* < .001, η^2^_p_ = .041]. Follow-up tests found that the three countries differed in liking for the café and the mall. The Swedish group (*M* = 5.5, *SD* = 1.07) had a higher preference for café’s than the Dutch [*M* = 5.0, *SD* = 1.56; *t*(493) = 2.40, *p* < .05] and Americans [*M* = 5.1, *SD* = 1.28; *t*(493) = 2.88, *p* <. 01], who did not differ from each other. For the mall the three groups all differed: the Dutch (*M* = 5.2, *SD* = 1.56) had higher ratings than the Americans [*M* = 4.7, *SD* = 1.54; *t*(493) = 3.82, *p* < .001], who had higher ratings than the Swedes [*M* = 4.3, *SD* = 1.62; *t*(493) = 2.17, *p* < .05].

With regard to Hypothesis 1b, country moderated restoration likelihood ratings for setting [*F*(6, 964) = 7.61, *p* < .001, η^2^_p_ = .045]. Likelihood of restoration was rated differently for parks and malls; the Swedes (*M* = 5.2, *SD* = 1.28) gave a lower rating to parks than the Americans [*M* = 5.6, *SD* = 1.28; *t*(493) = 2.47, *p* < .05], whereas the Dutch (*M* = 5.4, *SD* = 1.21) did not differ from either of the other groups. For the mall, the ratings differed between all three groups: the Dutch (*M* = 3.7, *SD* = 1.43) gave higher ratings than the Americans [*M* = 3.2, *SD* = 1.52; *t*(493) = 3.07, *p* < .01], who gave higher ratings than the Swedes [*M* = 2.4, *SD* = 1.20; *t*(493) = 4.50, *p* < .001].

With regard to Hypothesis 2a, country moderated the effect of company on preference [*F*(2, 484) = 3.17, *p* < .001, η^2^_p_ = .027]. The trend of lower preference for being alone versus in company was stronger in the Dutch group than in the other two. For likelihood of restoration (Hypotheses 2b) a similar effect did not appear (*p* > .50).

The interactive effect of company and setting on preference (Hypothesis3a) was in turn moderated by country [*F*(6, 966) = 2.29, *p* < .05, η^2^_p_ = .014]. Interaction effects per setting were found for the park and the café (*p*s < .05), not for the mall or the street (*p*s > .48). For the park setting the Americans proved to be indifferent to being alone or in company, while the Dutch and Swedes preferred being in company. For the café setting, the Dutch participants disliked being alone while the Americans and the Swedes were indifferent. For likelihood of restoration (Hypothesis 3b), there was also a Company x Setting x Country interaction effect. For the park setting the Americans differed from the others in that they saw greater likelihood for restoration when alone while the others saw greater restoration likelihood when in company. In the street setting the Swedes saw greater restoration likelihood when alone, while the other two were indifferent (*p*s < .05).

The interactive effect of attentional fatigue and setting on preference (Hypothesis 4) is also moderated by country [*F*(6, 966) = 3.28, *p* < .05, η^2^_p_ = .020]. There were specific interaction effects for two settings. For the park setting, Dutch participants expressed greater preference when fatigued, while for the Swedes the effect was the opposite, and the Americans were indifferent (p < .01). For the street setting, preferences were higher for Dutch and American participants who were alert while for the Swedes preferences were higher when fatigued (p < .05). For the café and mall setting, preferences were the same across nationalities.

For likelihood of restoration, effects of attentional fatigue by setting were also moderated by country [*F*(6, 964) = 4.20, *p* < .001, η^2^_p_ = .025]. Specific interaction effects per setting emerged for the park and the street setting. For the park, Dutch participants considered restoration more likely when imagining that they were fatigued, while the Swedes who were given the no-fatigue scenario conside this more likely and the Americans are indifferent (p < .01). For the street setting, the Swedes consider restoration more likely when fatigued, while the Dutch and Americans are indifferent (*p*s < .02).

Summarizing the effects of country, we conclude that several effects of our social context and antecedent condition manipulations, of the settings, and of their combinations, differ for participants in the three countries who participated in this study.

## Discussion

This study was undertaken with several objectives. The first and most important one was to improve understanding of urban options for restoration. Much leisure behavior actually takes place in urban settings, but the literature on restorative environments has relatively little to say about the restorative potential of behavior in many of these settings. In designing the present study, we followed an earlier study that looked at urban settings for restoration [31]. One specific goal originating from that earlier study was that we wanted to be more specific than previously in defining study settings in terms of the activity being performed as well as the environment to better see how preference and restoration likelihood ratings for these settings would be affected by social context and attentional fatigue. Our expectation was that, with increased behavioral specificity, the pattern of outcomes would also become more specific, and thereby clearer than in the previous study.

A second goal was to make comparisons across different countries. To date, there is only one cross-cultural study on restorative experiences framed in terms of attention restoration theory [55]. The focus of that study, however, was on developing an instrument to assess the restorative quality of different environments, not on the differential effects of settings in interaction with manipulations of attentional fatigue and social context.

These considerations led us to include in our experimental design four activity-environment combinations crossed with two different social contexts (absence or presence of a friend) and two levels of attentional fatigue (high or low), and to implement this design in the Netherlands, Sweden and the USA with roughly comparable methods and student samples.

Initial results of the tests guided by our hypotheses are that, in line with Hypothesis 1, the busy urban street received the lowest preference and restoration likelihood ratings of the four settings, with or without company, whether attentionally fatigued or alert, in all three countries. This may seem a rather trivial finding; however, given the frequent use of such a setting in studies of natural versus. urban differences in preferences and restorative effects, it is important that we clearly see here that the street setting is not representative of the range of different settings that can be distinguished within urban environments. Our results were also in line with Hypothesis 2a regarding a general preference for being in company compared to being alone. A corresponding effect was absent for restoration likelihood, counter to Hypothesis 2b. More important than the results bearing on Hypothesis 2, however, are the outcomes for Hypothesis 3 regarding the moderating effect of company on ratings of the different settings. The results for Hypothesis 3a qualify the outcomes bearing on Hypothesis 2a: as expected, the café and mall settings were preferred more when in company. In contrast, preferences for the park and the street were similar in the two company conditions. Restoration likelihood ratings for the four settings were not sensitive to the company variable, in contrast to Hypothesis 3b.

Attentional fatigue also proved to differentially influence preference for settings: preference for the park increased relative to that for a café or a mall, in line with Hypothesis 4. We found that the judged restoration likelihood for the four settings was not differentially sensitive to the need for restoration, an outcome for which we had not formulated predictions in advance.

Country of residence further conditioned many of the effects of the other variables. We were in fact surprised by the influence that country appeared to have, though its effects generally were not strong. This pervasive moderating role may seem surprising, as our samples come from societies that do not drastically differ from each other in important respects: all three samples are from Western, fairly affluent countries and made up of student populations, young, and predominantly female. Differences might be substantially larger when looking across a broader range of countries and continents. On the other hand, as we explained at the outset, the three countries–and the cities taken to represent them in this study–do differ in numerous ways relevant to the experience of the city. Cross-cultural studies that systematically and uniformly look for similarities and differences are needed to answer such questions. Answers seem not to be straightforward in pointing out cross-cultural differences: for example, preferences for urban green space in Asian cities seem to originate from needs similar to those in American and European cities (see [56]).

What do we learn from these results? This study extends a line of research in which different contextual and psychological variables have been investigated with regard to their effects on preferences for and the judged likelihood of restoration in different environments. Earlier studies found that attentional fatigue influenced the relative preference for natural and urban settings [10], that being in the company of a friend could boost preference for some settings and lower it for others [11], and that urban settings other than parks also supported expectations of restoration [31]. Many of the effects of those earlier studies are found again in this study, including the interactive effect of attentional fatigue and settings (especially street vs park) on preference as well as the effect of company. This combination of replication both in similar and more field-based research situations is encouraging, and it affirms the external validity of the findings.

We do of course acknowledge that our study has several limitations. First, our samples were ones of convenience. Across the three countries, our participants were similar in terms of a major determinant of restoration need, namely, occupation; however, we make no claim that our results are representative of the experience of all people in all cities in the three countries.

Second, the samples differed in some participant characteristics. The Swedish sample was somewhat older than the Dutch sample and the American sample. The Swedish sample also likely distinguished itself in terms of subject of study, in that it included students from diverse lines of study while the Dutch and American samples both consisted of introductory psychology students.

Third, the experiment was carried out somewhat differently in the different locations. Specifically, the Dutch study was preceded by an unrelated study and the randomization of the order of presentation of the four settings was done differently across the three locations.

We cannot state with certainty that our results have not been influenced by these differences in samples and method. But we do consider it unlikely that they played a major role; they are rather modest differences, and previous research with the kinds of general evaluative ratings collected here suggests that they are not sensitive to much more substantial methodological differences, such as whether ratings were collected on-site versus with photographic simulations [57, 58]. We can conclude that the settings, described in specific terms with regard to environment, activity, and immediate social context, are in fact appreciated differently by people with different restoration needs from different countries. This is the case both for ratings of preference and for likelihood of restoration. The conclusion should however be regarded as tentative with regard to the moderating role of the country of residence; it stands as a starting point for further research.We also must note some limitations with regard to comparisons of the present results to the results of earlier studies in this line of research. We consider it a strong point that some of the earlier studies used an experimental paradigm in which participants had to imagine being fatigued or not, being in the company of friends or alone, and being in different environments, while in other of the earlier studies actual fatigue was evoked [10, 12], and real company determined differential outcomes of a real walk in different environments in a manner consistent with the findings of the earlier scenario-based research [11, 13]. For example, Staats et al. [10] found that a difference in preference for a walk along streets in an urban center versus a walk in a forest was greater among those who imagined themselves in a condition of attentional fatigue versus no attentional fatigue. This interaction effect was found with Dutch students using environmental exemplars from the Netherlands and a scenario manipulation of attentional fatigue. A similar interaction was subsequently found with Swedish students using environmental exemplars from Sweden, but with a naturalistic manipulation of attentional fatigue, not scenarios [12]. Appreciation of such concurrence of results across studies with different methods, or the lack of concurrence, must be tempered by understanding that the studies encompassed in this line of research are not perfectly comparable. This line of research can benefit from further studies that seek to replicate and extend the current findings.

A related issue is how the findings obtained with preference and restoration likelihood measures relate to findings obtained with objective measures of directed attention capacity or other resources used in everyday coping. The extant literature provides multiple examples of congruence between results obtained with the different measures. For example, multiple experiments have used objective measures of attention and/or physiology to describe a restorative advantage of walking in or viewing a natural setting versus an urban street setting [46, 59, 60], in line with our finding of greater preference and restoration likelihood ratings for the urban park versus urban street setting. It is important to bear in mind, however, that objective measures useful for gauging actual restoration were employed in experiments that examined individuals on single, specific occasions, while the preference and restoration likelihood measures used in the present study and our earlier studies tap into the experiences that people have already had with restoration in different environments and bring that experience to bear on environments they might visit in a (future) hypothetical situation. It is not reasonable to expect that general ratings of preference and restoration likelihood will translate into comparable, objectively measurable outcomes on every single occasion a person might be observed in a given environment. Our measures reflect preferences and expectations that could lead a person in need of restoration into a particular environment; they do not imply a guarantee that restoration will occur. This issue aside, we note that the use of objective measures is relatively costly, and for that reason has historically constrained environmental sampling to a few comparison environments. Subtle differences between environments have therefore not been studied using objective measures, and comparisons of outcomes realized with different kinds of restorative activities that are typical for specific environments (i.e., activities-in-environments) have not even been approached using objective measures. We think that the measures we have used provide an economical approach that lays the foundation for experimentation with objective measures of restoration. For further discussion of this issue, see Hartig [23]. What utility do these results have in the end? We cannot go beyond the data that we have, which implies that we cannot fully explain the differences in effects found across the three samples. Our results nonetheless affirm earlier findings regarding the potential for a broader range of urban settings to serve restoration needs. Among other things, they also affirm the significance of the immediate social context and needs for attentional restoration as moderators of expectations of restoration in different settings. Not least, they can serve as an impetus for further research. We expect that new studies in which contextual and person characteristics are investigated still more systematically will yield results that are both scientifically interesting and practically relevant for urbanizing societies.
